# Using Automated HbA1c Testing to Detect Diabetes Mellitus in Orthopedic Inpatients and Its Effect on Outcomes

**DOI:** 10.1371/journal.pone.0168471

**Published:** 2017-01-06

**Authors:** Elif I. Ekinci, Alvin Kong, Leonid Churilov, Natalie Nanayakkara, Wei Ling Chiu, Priya Sumithran, Frida Djukiadmodjo, Erosha Premaratne, Elizabeth Owen-Jones, Graeme Kevin Hart, Raymond Robbins, Andrew Hardidge, Douglas Johnson, Scott T. Baker, Jeffrey D. Zajac

**Affiliations:** 1 University of Melbourne, Department of Medicine, Austin Health, Melbourne, Australia; 2 Department of Endocrinology, Austin Health, Melbourne, Australia; 3 Menzies School of Health Research, Darwin, Australia; 4 The Florey Institute of Neuroscience & Mental Health, Melbourne, Australia; 5 Austin Centre for Applied Clinical Informatics, Austin Health, Melbourne, Australia; 6 Department of Intensive Care, Austin Health, Melbourne, Australia; 7 Department of Orthopaedic Surgery, Austin Health, Melbourne, Australia; 8 Department of General Medicine, Austin Health, Melbourne, Australia; Virginia Commonwealth University, UNITED STATES

## Abstract

**Aims:**

The prevalence of diabetes is rising, and people with diabetes have higher rates of musculoskeletal-related comorbidities. HbA1c testing is a superior option for diabetes diagnosis in the inpatient setting. This study aimed to (i) demonstrate the feasibility of routine HbA1c testing to detect the presence of diabetes mellitus, (ii) to determine the prevalence of diabetes in orthopedic inpatients and (iii) to assess the association between diabetes and hospital outcomes and post-operative complications in orthopedic inpatients.

**Methods:**

All patients aged ≥54 years admitted to Austin Health between July 2013 and January 2014 had routine automated HbA1c measurements using automated clinical information systems (CERNER). Patients with HbA1c ≥6.5% were diagnosed with diabetes. Baseline demographic and clinical data were obtained from hospital records.

**Results:**

Of the 416 orthopedic inpatients included in this study, 22% (n = 93) were known to have diabetes, 4% (n = 15) had previously unrecognized diabetes and 74% (n = 308) did not have diabetes. Patients with diabetes had significantly higher Charlson comorbidity scores compared to patients without diabetes (median, IQR; 1 [0,2] vs 0 [0,0], *p*<0.001). After adjusting for age, gender, comorbidity score and estimated glomerular filtration rate, no significant differences in the length of stay (IRR = 0.92; 95%CI: 0.79–1.07; *p* = 0.280), rates of intensive care unit admission (OR = 1.04; 95%CI: 0.42–2.60, *p* = 0.934), 6-month mortality (OR = 0.52; 95%CI: 0.17–1.60, *p* = 0.252), 6-month hospital readmission (OR = 0.93; 95%CI: 0.46–1.87; *p* = 0.828) or any post-operative complications (OR = 0.98; 95%CI: 0.53–1.80; *p* = 0.944) were observed between patients with and without diabetes.

**Conclusions:**

Routine HbA1c measurement using CERNER allows for rapid identification of inpatients admitted with diabetes. More than one in four patients admitted to a tertiary hospital orthopedic ward have diabetes. No statistically significant differences in the rates of hospital outcomes and post-operative complications were identified between patients with and without diabetes.

## Introduction

The prevalence of diabetes mellitus worldwide is rising [1]. In Australia, the estimated prevalence of diabetes is 7%, and has doubled since the 1980s [2]. People with diabetes are more likely to have chronic musculoskeletal comorbidities [3]. However, the prevalence of diabetes, both known and unrecognized, among orthopedic inpatients within a tertiary hospital has rarely been studied [4, 5, 6, 7].

In surgical patients, a history of diabetes is associated with increased mortality and poorer outcomes, such as prolonged length of hospital stay, hospital readmission, intensive care unit (ICU) admission, and post-operative complications [8]. However, there is limited and conflicting evidence regarding outcomes specifically in orthopedic patients. Some studies [7, 9, 10, 11] have reported a higher likelihood of poor outcomes among inpatients with diabetes, whilst others [12, 13] reported no increase in risk.

HbA1c (glycosylated hemoglobin) testing is a suitable option for diabetes detection in hospital inpatients, as it is unaffected by factors such as fasting status, glucocorticoid use and stress hyperglycemia. Both the American Diabetes Association (ADA) and the International Expert Committee have endorsed HbA1c as the diagnostic test of choice for diabetes mellitus, using a threshold of 6.5% (48mmol/mol) [14, 15].

Previous studies reporting the use of computerized clinical information systems for the identification and treatment of patients with diabetes have been conducted in the outpatient setting, and have relied on retrospective and historical data, rather than real-time biochemical measures [16, 17]. Automated inpatient HbA1c testing is currently not routine practice. The aims of this prospective study were (i) to demonstrate the feasibility of using routine HbA1c testing via automated clinical information systems for detection of diabetes, (ii) to determine the prevalence of known and unrecognized diabetes in orthopedic inpatients and (iii) to investigate the association of diabetes with hospital outcomes and post-operative complications.

## Materials and Methods

### Patients

In this prospective observational cohort study, we studied all patients aged ≥54 years admitted under the Austin Health orthopedic unit during the period 17 July 2013 to 17 January 2014. Patients with multiple admissions during the study period were assessed on the basis of the first admission.

Pre-specified de-identified background demographics (age and gender), admission diagnosis, joint type (hip, knee, spine, other), clinical characteristics (medical/surgical history, medications, treatments administered), outcome of episode (length of stay, ICU admission, 6-month mortality and 6-month readmission) and biochemical laboratory values (HbA1c, hemoglobin and serum creatinine levels) were extracted from relevant hospital databases. “Mortality” was established if patient death: (i) had occurred during the hospital admission or (ii) was reported to the hospital during the 6-month study period. “Readmission” was defined as readmission to this hospital during the 6-month study period. Estimated glomerular filtration rate (eGFR) was calculated based on the CKD-EPI formula [18] using extracted data (age, gender and creatinine). Post-operative inpatient complication data, including any complication, infection (surgical site infection, peri-prosthetic joint infection, urinary tract infection, pneumonia and bacteremia/sepsis), venous thromboembolism (deep vein thrombosis and pulmonary embolism), acute renal failure, delirium and anemia, were identified through methodical manual audit of the hospital medical records.

### Procedures & Measurements

**Fig 1:**
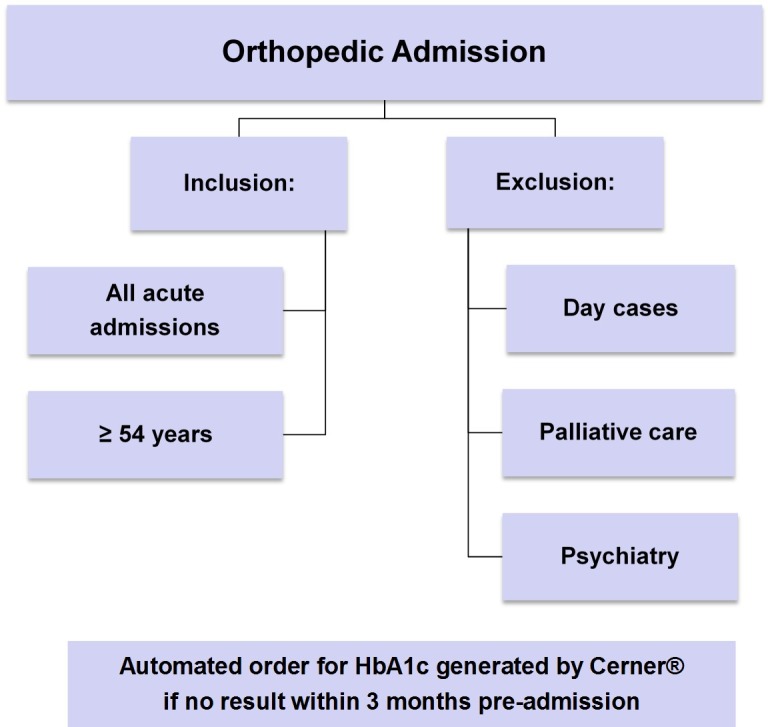
Criteria for automated generation of HbA1c Test Request on Cerner®.

As described in detail previously [19], during the six-month study period, all eligible participants underwent HbA1c testing on admission as part of routine clinical care. This was coordinated by an automated pathology test order through the Cerner Millennium® Clinical Information System, by which a request for an HbA1c was automatically generated if the inclusion criteria (age ≥54 years, acute hospital admission and no recorded HbA1c result within 3 months pre-admission) were satisfied (Fig 1) [19]. All HbA1c results were reported and accessible by treating clinicians through the Cerner Millennium® software interface.

Whole blood was obtained from eligible patients in ethylenediaminetetraacetic acid (EDTA) tubes. HbA1C was measured by turbidimetric inhibition immunoassay (TINIA) on Cobas Integra 800 (Roche Diagnostics, Mannheim, Germany), standardized to the IFCC reference method with a between-run co-efficient of variation of 2.5% for HbA1c 5.6% (30mmol/mol) and 1.5% for HbA1c 9.7% (83mmol/mol).

Patients were categorized into three groups according to hospital medical records and HbA1c level: (i) “Known diabetes” (diabetes diagnosis documented in the medical record with International Classification of Disease 10 Australian modification [ICD-10-AM] codes E10-14); (ii) “Unrecognized diabetes” (no diabetes diagnosis documented in the medical record and HbA1c ≥6.5% [48 mmol/mol]) and (iii) “No diabetes” (no diabetes diagnosis documented in the medical record and HbA1c <6.5% [48 mmol/mol]) [19]. A second confirmatory test was recommended in the discharge summary for patients with previously unrecognized diabetes. Data from patients with “known diabetes” and “unrecognized diabetes” were amalgamated to form a combined group (“patients with diabetes”) for outcome analyses.

Information regarding comorbidities was extracted from each patient’s hospital medical record for calculation of a validated adaptation of the Charlson index score, calculated from ICD-10-AM codes [20]. Given that diabetes status was considered a separate variable, the calculated Charlson comorbidity score was modified to exclude diabetes. To account for the possibility of disease coding underestimating the true prevalence of diabetes and co-morbidities, medical records of 20% of the sample were manually audited, yielding similar results.

### Ethics

The study was approved by the Austin Health Human Research Ethics Committee, who waived the need for informed consent for a planned practice change agreed to by hospital senior medical staff members as part of the Austin Health Diabetes Discovery Initiative.

### Statistical Analysis

Patient groups were compared with respect to baseline characteristics, length of stay, intensive care unit (ICU) admission, inpatient mortality, hospital readmission at 6 months and post-operative complication rates (any complication, infection, venous thromboembolism, acute renal failure, delirium and anemia).

Continuous explanatory variables are presented as medians with interquartile ranges (IQR), and analyzed using Wilcoxon rank-sum tests (non-parametric distribution). Categorical explanatory variables are reported as percentages and analyzed using Fisher’s exact test. Association between diabetes status (with or without) and hospital outcomes adjusted for age, gender, Charlson comorbidity score (excluding diabetes), and estimated glomerular filtration rate were investigated using random-effects regression models (negative binomial regression for the length of stay and logistic regression for ICU admission, 6-month mortality, 6-month readmission, post-operative complications and its subtypes) with joint type treated as a random effect. Due to the heterogeneity of the orthopedic inpatient population, an additional exploratory subgroup analysis of outcomes was conducted in patients admitted for hip joint surgery, who comprised the largest subgroup of orthopedic patients. Given the exploratory nature of this study, no adjustment for multiple testing was undertaken. All *p*-values were calculated from two-tailed tests of statistical significance with the threshold being 5%.

## Results

### Patient Characteristics

**Fig 2:**
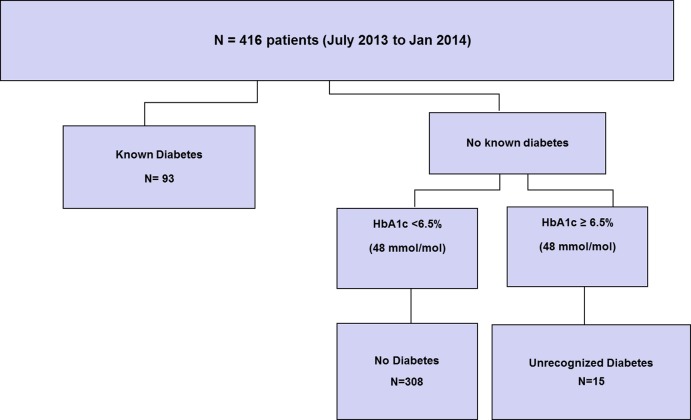
Study Profile.

**Fig 3:**
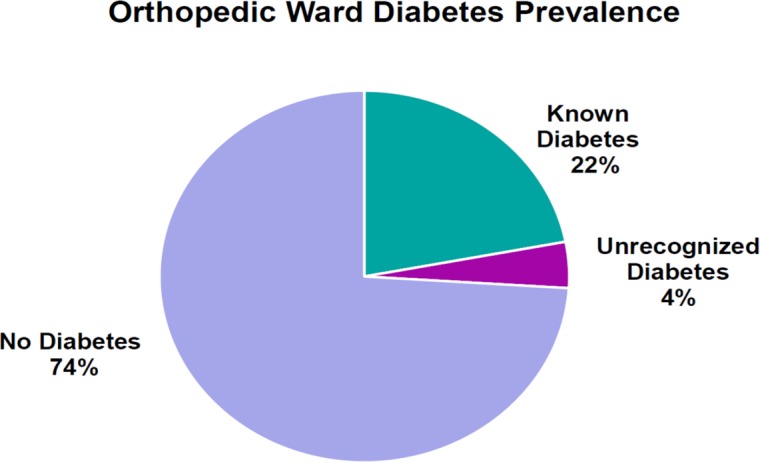
Prevalence of known, unrecognized and no diabetes in inpatients ≥54 years.

A total of 416 consecutive orthopedic inpatients aged ≥54 years had routine automated HbA1c testing using CERNER and were included in this prospective observational study (Fig 2). The prevalence of diabetes mellitus in the sample population was 26% (n = 108), comprising 22% (n = 93) with known diabetes, and 4% (n = 15) with previously unrecognized diabetes (Fig 3).

The baseline characteristics of orthopedic inpatients with and without diabetes are shown in Table 1. No significant differences in age, gender distribution, baseline hemoglobin, eGFR, and proportion of patients who underwent operative interventions were observed between the two groups. However, patients with diabetes had significantly more comorbid medical conditions compared to those without diabetes, as evidenced by the higher Charlson score (median, IQR; 1 [0,2] vs 0 [0,0], *p*<0.001). At Austin Health, routine data collected on ethnicity is regarding whether a person is Indigenous, that is of Aboriginal and Torres Strait Islander background. Out of the 416 patients, 405 were non Indigenous, that is neither Aboriginal or of Torres Strait Islander origin. 11 patients were coded as unable to be asked the question, 2 in the no diabetes group and 9 in the no diabetes group (p = 0.58). Therefore there were no differences in Indigenous status between the groups.

**Table 1:** Baseline characteristics by diabetes status in all orthopedic patients.

Clinical Characteristics	N	Diabetes	No Diabetes	p-value^¶^
Number (%)	416	26%	74%	N/A
Male (%)	416	37.0%	34.7%	0.726
Age (years)	416	74 (63,79)	75 (65,85)	0.092
HbA1c (%)	416	6.9 (6.4,7.7)	5.7 (5.4,5.9)	<0.001
HbA1c (mmol/mol)	416	51.9 (46.4,60.7)	38.8(35.5,41.0)	<0.001
Hemoglobin (g/L)	413	119 (105,132)	117 (104,127)	0.299
CKD-EPI eGFR (ml/min/1.73m^2^)	413	67 (48,87)	74 (57,87)	0.108
Charlson Comorbidity Score^#^	416	1 (0,2)	0 (0,0)	<0.001
Operative* (%)	416	77.8%	82.1%	0.321
Elective^^^ (%)	337	40.5%	30.4%	0.108

Categorical explanatory variables are reported as percentages and continuous explanatory variables are summarized as medians with interquartile ranges in parenthesis.

^¶^ p-values were determined by Fisher’s exact test for categorical variables and Wilcoxon rank-sum test.

^# ^Charlson comorbidity score = a validated method of weighting chronic medical conditions (the score for diabetes was excluded as diabetes is included as a separate variable)

* Operative = admissions involving an orthopedic operative intervention.

^ Elective = admissions scheduled in advance and not constituting a medical emergency.

Abbreviations: HbA1c = Glycosylated hemoglobin (a measure of long term glycemic status), CKD-EPI = Chronic Kidney Disease-Epidemiology Collaboration equation, eGFR = estimated glomerular filtration rate.

### Patient Outcomes

In unadjusted analyses of 6-month outcomes, there were no significant differences between patients with and without diabetes in length of stay, (median, IQR; 5.5 [3.3,10.0] days for patients with diabetes vs 7.0 [4.0,12.0] days for patients without diabetes, *p* = 0.34), proportions of ICU admissions (7.4% for patients with diabetes vs 5.8% for patients without diabetes, *p* = 0.64), 6-month mortality (4.6% for patients with diabetes vs 6.8% for patients without diabetes, *p* = 0.50) and readmissions (12.0% for patients with diabetes vs 12.3% for patients without diabetes, *p* = 1.00). Similarly, there were no significant differences between patients with and without diabetes in rates of post-operative complications (any complication 32.1% for patients with diabetes vs 34.8% for patients without diabetes, *p* = 0.69; acute renal failure 7.1% for patients with diabetes vs 3.6% for patients without diabetes, *p* = 0.22; infection: 8.3% for patients with diabetes vs 11.1% for patients without diabetes, *p* = 0.54; venous thromboembolism: 0% for patients with diabetes vs 1.6% for patients without diabetes, *p* = 0.58; delirium: 9.1% for patients with diabetes vs 7.1% for patients without diabetes, *p* = 0.66; anemia: 22.9% for patients with diabetes vs 25% for patients without diabetes, *p* = 0.77).

The multivariate analyses examining the independent association of diabetes with outcomes are shown in Table 2. After adjusting for age, gender, comorbidity score and eGFR, with joint type treated as a random effect, no significant differences in hospital outcomes or any post-operative complications were detected between patients with and without diabetes.

**Table 2:** Adjusted outcome data comparing all orthopedic patients (n = 416) with diabetes versus patients without diabetes.

Hospital Outcomes*^#^	**IRR/OR**^**^**^ **(95% CI)**	^¶^**p-value**
Length of Stay (days, IRR)	0.92 (0.79–1.07)	0.280
ICU Admission (OR)	1.04 (0.42–2.60)	0.934
6-Month Mortality (OR)	0.52 (0.17–1.60)	0.252
6-Month Readmission (OR)	0.93 (0.46–1.87)	0.828
Post-operative Inpatient Complications*^#^	**IRR/OR**^**^**^ **(95% CI)**	^¶^**p-value**
Any Complication (OR)	0.98 (0.53–1.80)	0.944
Acute Renal Failure (OR)	2.06 (0.58–7.32)	0.266
Infection (OR)	0.84 (0.34–2.10)	0.711
Venous Thromboembolism (OR)	N/A	N/A
Delirium (OR)	1.34 (0.45–3.99)	0.602
Anemia (OR)	1.36 (0.70–2.61)	0.364

* Adjusted for age, gender, Charlson comorbidity score^#^ and CKD-EPI eGFR, with joint type treated as a random effect.

^¶ ^p-values were determined by Fisher’s exact test for categorical variables and Wilcoxon rank-sum test.

# Charlson comorbidity score = a validated method of weighting chronic medical conditions (the score for diabetes was excluded as diabetes is included as a separate variable)

^ IRR/OR derived by comparison between patients with diabetes (known and unrecognized) and patients without diabetes (reference category).

Abbreviations: ICU = intensive care unit, IRR = incidence rate ratio (applicable to continuous variables), OR = odds ratio (applicable to categorical variables).

Similarly, in the multivariate analysis of the subgroup of orthopedic inpatients admitted for hip joint surgery (n = 142), after adjusting for age, gender, comorbidity score and eGFR, there were no significant differences in any the aforementioned hospital outcomes and post-operative complications between patients with and without diabetes (Table 3).

**Table 3:** Outcome data comparing orthopedic Hip surgical patients (n = 142) with diabetes versus patients without diabetes.

Hospital Outcomes*^#^	**IRR/OR**^**^**^ **(95% CI)**	^¶^**p-value**
Length of Stay (days, IRR)	0.96 (0.75–1.23)	0.735
ICU Admission (OR)	N/A	N/A
Inpatient Mortality (OR)	0.33 (0.06–1.80)	0.202
Hospital Readmission at 6 months (OR)	0.94 (0.26–3.40)	0.930
Post-operative Inpatient Complications*^#^	**IRR/OR**^**^**^ **(95% CI)**	^¶^**p-value**
Any Complication (OR)	1.07 (0.40–2.89)	0.893
Acute Renal Failure (OR)	1.85 (0.30–11.27)	0.506
Infection (OR)	2.13 (0.68–6.70)	0.194
Venous Thromboembolism (OR)	N/A	N/A
Delirium (OR)	1.06 (0.28–3.95)	0.937
Anemia (OR)	1.81 (0.66–4.95)	0.250

* Adjusted for age, gender, Charlson comorbidity score^#^ and CKD-EPI eGFR.

^¶ ^p-values were determined by Fisher’s exact test for categorical variables and Wilcoxon rank-sum test.

# Charlson comorbidity score = a validated method of weighting chronic medical conditions (the score for diabetes was excluded as diabetes is included as a separate variable)

^ IRR/OR derived by comparison between patients with diabetes (known and unrecognized) and patients without diabetes (reference category).

Abbreviations: ICU = intensive care unit, IRR = incidence rate ratio (applicable to continuous variables), OR = odds ratio (applicable to categorical variables).

## Discussion

The present study demonstrated the feasibility of utilizing automated clinical information systems to automatically order HbA1c measurement for identification of inpatients with diabetes. We also investigated the burden of diabetes and its association with hospital outcomes in the orthopedic wards of a tertiary hospital. Amongst 416 orthopedic inpatients above the age of 54 years, 22% had known diabetes and 4% had previously unrecognized diabetes. Furthermore, we examined the associations between the presence of diabetes and patient outcomes within the study period of 6 months and found that there were no differences between those with and without diabetes.

For many years, the diagnosis of diabetes has been made using criteria based on the laboratory measurement of blood glucose levels [14]. This is associated with some inherent issues, such as the requirement for fasting, and the potential confounding effects of glucocorticoid use and stress hyperglycemia, thereby rendering it a suboptimal tool for diabetes detection among inpatients. In contrast, HbA1c has been recommended as the preferred case-finding tool in the hospital setting, as it is able to distinguish between previously unrecognized diabetes and transient stress hyperglycemia. Although HbA1c values are affected by diseases of erythrocytes, such as hemoglobinopathies, these are uncommon and can be accommodated by alternative tests [21]. Moreover, previous research has supported the implementation of routine admission HbA1c testing as a means to identify and manage at-risk patients appropriately [22].

Electronic health records and programmable clinical informatics have been increasingly utilized for the purpose of detecting and treating inpatients at risk of various conditions. For instance, Herasevich and colleagues demonstrated the feasibility of “sepsis sniffer”, an automated electronic screening tool, to identify inpatients with severe sepsis [23]. Examples in other settings include gastrointestinal malignancy [24] and chronic kidney disease [25]. To our knowledge, the present study is the first to demonstrate the successful implementation of admission HbA1c testing as part of routine clinical care in orthopedic inpatients. Using a combination of electronic medical records and real-time biochemical measures, we have shown the feasibility of this approach for detection of diabetes in inpatients, representing a window of opportunity to engage with people at high risk whose diabetes may otherwise remain undiagnosed. Furthermore, the automatic inclusion of the diabetes status in the patient’s discharge summary addresses a current deficiency in healthcare transition from the orthopedic wards to the community setting [13, 26].

There is evidence that the diabetes prevalence is rising worldwide, and this is reflected amongst patients undergoing joint replacement surgeries [4, 5]. In the present study of 416 orthopedic inpatients, our identification of 22% with known diabetes and 4% with previously unrecognized diabetes is similar to the findings of a South Korean study of 173 patients undergoing elective ankle replacement surgery, in whom known and unrecognized diabetes respectively were identified in 22% and 2.9% of patients [27]. In contrast, in another study of 275 hip, knee and spinal surgical patients, it was found that 12% and 3% had known and unrecognized diabetes, respectively [26]. These studies were limited by their smaller sample sizes, and the use of plasma glucose levels or retrospective chart review to classify diabetes status. Moreover, both of these studies were conducted in specific joint cohorts and may not represent the true burden of diabetes within the broader population of orthopedic inpatients. One other study in which medical records of all patients who had orthopedic surgery over a 2-year period in a single Chinese hospital were retrospectively examined [6] reported that 4.7% of orthopedic inpatients had known diabetes. In this study, diabetes prevalence was not the primary outcome and was not determined from objective biochemical measures, such as HbA1c in the present study. As a result, the prevalence of unrecognized diabetes could not be determined. These limitations highlight the need for further research within the orthopedic ward setting.

It has been shown that a diagnosis of diabetes is associated with increased surgical inpatient mortality [8] and morbidity [9], such as length of stay, readmission and ICU admission. In our study, after adjusting for several confounding covariates including Charlson comorbidity scores, no significant difference in 6-month mortality was detected between patients with and without diabetes. There is variation in the literature regarding the effect of diabetes on mortality following orthopedic surgery. In a recent retrospective study of 66,485 patients undergoing shoulder replacement surgery, diabetes was found to independently increase the odds of mortality (adjusted odds ratio for in-hospital death 2.1; 95% CI 1.4–3.4; *P* < 0.001) [28]. In contrast, another study of 197,461 spinal surgical patients reported that diabetes was not independently associated with increased mortality [12]. It is worth noting that these studies only examined inpatient deaths and did not collect mortality data after discharge. Others have reported a reduced risk of in-hospital mortality, but poorer 5-year survival after vascular surgery in people with diabetes [29]. While mortality data in our study was determined over a period of 6 months, patient death occurring after discharge was only recorded if this was communicated to the hospital, thus potentially underestimating the true mortality in our sample population.

We also did not find a significant association between diabetes and other outcomes such as length of stay, 6-month readmission and ICU admission. The literature regarding the effects of diabetes on length of stay is not consistent. Several studies investigating joint replacement surgical patients have found that diabetes significantly increased length of stay [28, 30], while others have disputed this association [13, 31]. Of note, in contrast to many studies which reported positive findings, in the present study, the length of stay was analyzed with median values, which better reflect the non-parametric nature of this outcome variable than mean values. Furthermore, a negative binomial regression analysis was undertaken for the length of stay in the current study to take into account the highly skewed distribution of this outcome. There is limited evidence with regards to the effect of diabetes on readmission and ICU admission rates in orthopedic patients. There is only one other study, which demonstrated that controlled diabetes (defined as HbA1c <7%), but not uncontrolled diabetes, may be associated with slightly increased odds for readmission within 12 months of total knee arthroplasty [32].

It has been shown that diabetes increases the risk of post-operative complications in the surgical setting [8]. However, limited and contradictory evidence exists regarding orthopedic surgery, with some studies showing that a history of diabetes increases post-operative complication risk [33], while other studies do not [34]. After controlling for several confounding variables with joint type treated as a random effect, we observed no significant associations between diabetes diagnosis and any of the examined post-operative complications, including infection, venous thromboembolism, acute renal failure, delirium and anemia. There are several potential reasons which may account for this finding. Firstly, all of the measured post-operative complications were rare in our study, which was conducted in a large metropolitan tertiary health service. Furthermore, the diabetes cohort in the present study had well-controlled diabetes (median HbA1c 6.9% [IQR 6.4, 7.7]), which is likely to have mitigated the risks associated with the diabetes in surgical settings. Finally, in keeping with other studies [6, 12], our study design did not allow us to monitor for any post-operative complications occurring after hospital discharge.

Our study has some other limitations. Firstly, in comparison to other multi-center or database-related studies, our sample size was relatively small (n = 416) and heterogeneous, with a short follow-up period of 6 months, and included only patients aged 54 years and above. We did not find differences in outcomes between those with and without diabetes (identified using HbA1c). This may be related to the heterogeneity of the study population and surgical procedures performed, and the relatively small size of the study population. Studies with greater numbers may be necessary to determine if there are differences in outcomes between people with and without diabetes, including those with newly diagnosed diabetes. On account of the small number of patients with diabetes, analyses of outcomes according to level of glycemic control were not conducted. However, our study is the first to demonstrate the feasibility of utilizing real-time HbA1c measurements for case-finding purposes, thereby allowing a more accurate identification of diabetes in the inpatient setting. Furthermore, this study offers some insight into the effects of diabetes on a variety of important outcomes, which have not previously been examined in this context. As the present study aimed to investigate the outcomes of orthopedic patients in a tertiary center, participants receiving both surgical and conservative (non-surgical) care were included in the analysis of hospital outcomes. As surgery does impose significant physiological stress, patients managed conservatively may not have the same level of risk as surgical patients. However, in contrast to many previous studies, this study does demonstrate the true diabetes prevalence within the orthopedic wards, which is pertinent to workforce planning regarding diabetes management and risk minimization in this setting. Another limitation is that we did not have data on the glucose readings or levels of physical activity or weight gain in the post-operative period which may have influenced glycemic control and therefore outcomes in people with diabetes, including those who were newly diagnosed.

In summary, we have demonstrated the feasibility of routine HbA1c measurement using CERNER used for real time case-finding of patients with diabetes. More than one in four patients above the age of 54 years admitted to the orthopedic wards of a tertiary health service have diabetes. In this study, patients with diabetes did not have a higher risk of adverse hospital outcomes and post-operative complications compared to those without diabetes. Future studies with larger cohorts and longer follow-up time are required to provide further insight into the impact of diabetes on orthopedic inpatient outcomes.

## Abbreviations

HbA1c: glycosylated hemoglobin
IQR: interquartile range
IRR: incidence rate ratio
OR: odds ratio
ADA: American Diabetes Association
ICU: intensive care unit
eGFR: estimated glomerular filtration rate
CKD-EPI: Chronic Kidney Disease-Epidemiology Collaboration equation
ICD: International Classification of Disease
