# Correction: Using Automated HbA1c Testing to Detect Diabetes Mellitus in Orthopedic Inpatients and Its Effect on Outcomes

**DOI:** 10.1371/journal.pone.0172380

**Published:** 2017-02-13

**Authors:** Elif I. Ekinci, Alvin Kong, Leonid Churilov, Natalie Nanayakkara, Wei Ling Chiu, Priya Sumithran, Frida Djukiadmodjo, Erosha Premaratne, Elizabeth Owen-Jones, Graeme Keith Hart, Raymond Robbins, Andrew Hardidge, Douglas Johnson, Scott T. Baker, Jeffrey D. Zajac

The tenth author’s name is spelled incorrectly. The correct name is: Graeme Keith Hart.
